# Understanding the impact of COVID-19 on comorbid depression, anxiety and eating disorders in adolescent psychiatric inpatients: a network analysis

**DOI:** 10.1186/s13034-025-00899-0

**Published:** 2025-04-23

**Authors:** Charlotte M. K. Milewczyk, Martin Holtmann, Tanja Legenbauer, Laura M. Derks

**Affiliations:** Department for Child and Adolescent Psychiatry, Psychosomatic and Psychotherapy, LWL University Hospital of the Ruhr University Bochum, Heithofer Allee 64, 59071 Hamm, Germany

**Keywords:** Depression, Anxiety, Eating disorder, Comorbidity, Adolescence, COVID-19, Network analysis

## Abstract

**Background:**

Many studies have aimed to understand the impact of the COVID-19 pandemic on mental health. However, less research has focused on the changes in symptom clusters of comorbid disorders. To understand the impact of the COVID-19 pandemic it is necessary to evaluate the relationships between symptoms of comorbid disorders. This was the first study to compare comorbidity networks of depression, anxiety and eating disorder (ED) symptoms to investigate the overall connectivity of symptoms before and during the onset of the pandemic.

**Methods:**

Self-report questionnaire data from 1361 adolescent psychiatric inpatients (*M*_*age*_ = 15.32, *SD* = 1.47) were used for this study. A network analysis was conducted including 52 questionnaire items of depression, anxiety and eating disorder to identify and compare core symptoms and bridge symptoms in a pre and a peri pandemic sample.

**Results:**

A significantly higher network density and overall connectivity were found in the peri pandemic sample. Links between feelings of failure in the depression cluster and worry what other people think in the anxiety cluster as well as between difficulties getting rid of bad/ silly thoughts in the anxiety cluster and suicidal thoughts in the depression cluster emerged as the strongest pathways in both networks. Body image disturbance emerged as the strongest bridge symptom for eating disorders in both networks. There were no significant differences in the most prominent core and bridge symptoms between the networks, indicating a high stability of core symptoms and pathways across circumstances.

**Conclusions:**

Our findings suggest a multidimensional relationship between symptoms of depression, anxiety, and eating disorders. The persistence of symptom pathways after the onset of the pandemic implies that these pathways may be responsible for the occurrence of comorbidity and should be primary targets of psychotherapy for affected patients. Addressing core and bridge symptoms in the therapy of comorbid disorders should be a priority and may be more effective than conventional treatment strategies.

**Supplementary Information:**

The online version contains supplementary material available at 10.1186/s13034-025-00899-0.

## Introduction

Depression, anxiety and eating disorders (EDs) are among the most common mental disorders in children and adolescents [1, 2]. Moreover, they show considerable comorbidity rates [3–5]. For example, studies show that over 80% of people diagnosed with ED also display characteristics of an anxiety disorder [5, 6], and over 60% of adolescents with major depressive disorder also meet the criteria for a comorbid anxiety disorder and vice versa [4].

The onset of the COVID-19 (Corona Virus Disease 2019) pandemic, along with corresponding infection control measures such as mobility restrictions and lockdowns enabled from March 2020, has had a substantial impact on youth populations’ mental health. Research showed that 75% of children and adolescents reported feeling troubled during the initial phase of the pandemic. The prevalence of psychological disorders has nearly doubled during this period in adolescents [7]. A significant increase was observed in the prevalence of anxiety disorder, eating disorders and depression in particular [8]. Analyses found a pooled 31% prevalence of both depressive and anxiety symptoms in children and adolescents [9]. Similarly, the rate in hospitalization of eating disorder patients has almost doubled in the period from 2020 to 2021 [10].

In addition to the rise in prevalence, studies detected an increase in symptom severity among patients with pre-existing mental health conditions [11–14]. For instance, adolescents with pre-existing ED experienced a pronounced exacerbation of ED symptoms as well as increased levels of anxiety and depression symptoms throughout the first waves of the pandemic [13]. Moreover, recent findings suggest that not only have individual diseases been affected by the onset of the pandemic, but also symptom relationships between comorbid disorders. It is assumed that a higher symptom severity is associated with a stronger interrelationship between symptoms [15]. These findings indicate that the onset of the pandemic might have led to higher correlations between symptoms of comorbid disorders.

To understand the impact of the COVID-19 pandemic on comorbidity it is necessary to evaluate which aspects of a condition increase relationships between different psychopathological conditions. Network analysis is considered a suitable methodological framework to investigate the direct interaction of symptoms and meaning of those relationships for the occurrence of comorbidity.

### Network theory

The network theory approach in psychopathology aims to shed light on the relationship between various aspects of mental disorders. Unlike the conventional assumption that symptoms result independently from a common cause– the underlying condition -, network theory suggests that mental illness can be viewed as a complex system of symptoms [16]. It is proposed that symptoms are functionally interrelated elements which have the ability to mutually activate, deactivate and maintain each other [16–18]. Consequently, occurrence, severity and duration of comorbidity depend on direct relationships between the symptoms of various disorders [17]. For example, depression is presumed to emerge from interactions among symptoms such as concentration problems leading to sleep deprivation resulting in fatigue rather than those symptoms being attributed to an underlying condition of depression [16].

Psychopathological networks consist of *nodes*, which represent symptoms, and *edges*, which represent the relationship between symptoms. Network analysis allows the display of a network graphically. A thicker *edge* symbolizes a stronger connection between two *nodes.* Disorders that are independent of each other would present themselves as separate networks of symptoms; symptoms of one disorder would have no or only sparse connections (i.e. edges) with symptoms of the other disorder. In contrast, networks of comorbid diseases would be linked to each other; symptoms of one disorder would have meaningful connections with symptoms of the other disorder [17, 19]. A useful parameter for understanding symptom relationships through network analysis is node centrality. Network analysis allows to detect which nodes within a network function as core symptoms of a disorder. According to network theory, a core symptom has numerous connections within a network and once activated, this node is most probable to trigger the activation of other symptoms across the network [16, 18]. Symptoms of one disorder with especially strong connections to symptoms of the other disorder can be regarded as bridge symptoms. For example, a symptom (e.g., rumination) in one cluster (e.g., depression) cannot only activate symptoms of that same cluster (e.g., depressed mood) but also function as a bridge to one or more symptom clusters of different disorders (e.g., panic attacks within anxiety disorder might be a bridge to depressive disorder). That means that patients with a certain symptom which has been detected as a bridge symptom are more likely to experience the activation of a comorbid network. Bridge symptoms are not necessarily symptoms that are most specific to the disorder. It is therefore essential to not only identify core symptoms but also bridge symptoms using network analysis, as otherwise the pathways responsible for comorbidity may remain hidden [18].

Furthermore, network analysis can be applied to compare data of different populations [17, 19]. The Network Comparison Test can be utilized to compare two or more networks regarding the way the symptoms within each network are connected (network structure) and regarding changes in the sum of all network edges displaying network density (global strength) [20].

In sum, the network approach presents the opportunity to uncover fresh perspectives as the investigation of a symptom network can unveil relational as well as structural characteristics of a disorder. Identifying non-obvious connections between symptoms, the network analysis approach allows to point out targets for intervention [18].

### Present study

While there are a few studies in which network analysis has been employed to study relationships among depression, anxiety, and ED [21], no attempt has yet been made to compare combined depression/anxiety/ED networks before and during the onset of the COVID-19 pandemic. The primary aim of the present study was to investigate the changes in network density, as assessed through the Network Comparison Test [20] to understand the impact of COVID-19 on comorbid networks. We hypothesize that the increase of symptom severity due to pandemic events has led to a stronger overall connectivity (global strength) during the pandemic (peri pandemic network) compared to before the pandemic (pre pandemic network).

The secondary objective of this study was to determine central and bridge symptoms of the networks. Thus, we conducted exploratory analysis of the network of ED, depression and anxiety to highlight particularly central symptoms within networks. We aim to focus on symptoms that form bridges between conditions, potentially making the greatest contribution to the occurrence and maintenance of comorbid disorders. The evaluation of bridge symptoms is essential and holds promise for enhancing interventions in the context of comorbidity. We further investigated if there were significant changes in (bridge) centrality between the pre and peri pandemic network.

## Materials and methods

### Participants

For the present study, data from 1401 psychiatric inpatients of the LWL-University Hospital for Child and Adolescent Psychiatry Hamm in Germany was used. Shortly after admission, all patients filled in a self-report questionnaire battery as part of a routine diagnostic assessment. Of the 1401 patients, 29 were excluded due to a diagnosis of psychotic disorders that could have impaired filling in the questionnaires. Participants were separated into two groups according to the COVID-19 pandemic status. Patients who were admitted before the outbreak of the pandemic (01/01/2019–15/03/2020) were assigned to the first group (pre COVID-19). Patients who were assessed between 30/03/2020 and 23/02/2022 were assigned to the second group (peri COVID-19). A further 11 patients who filled in the questionnaires during the two weeks between 16/03/2020 (official start of the first wave of COVID-19 and first corresponding measures in Germany) and 29/03/2020 were excluded from the study because several questionnaire items covered time periods both before and after the official onset of the pandemic. Therefore, those patients could not be properly assigned to the first or second group. To center our investigation on the influence of COVID-19 on psychological symptoms, we chose not to include data from patients admitted after the onset of the conflict in Ukraine as we could not definitively rule out an additional impact on the patients’ condition. The final pre pandemic sample consisted of 596 participants and the final peri pandemic sample consisted of 765 participants.

### Measures

#### PHQ-9

The German version of the Patient Health Questionnaire (PHQ-9 [22]), was used to measure depressive symptoms in the current sample. Its nine items refer to the Diagnostic and Statistical Manual of Mental Disorders IV (DSM-IV) diagnostic criteria for Major Depressive Disorder. The self-report measures use a 4-point Likert scale, ranging from 0 (“not at all”) to 3 (“nearly every day”) to assess the presence of depressive symptoms during the previous two-week period.

The PHQ-9 is considered a reliable, efficient and widely accepted diagnostic instrument for the assessment of depressive symptoms in both adults [23] and adolescents [24]. Cronbach’s α for the PHQ-9 in the current study was 0.87 for the total score.

#### SCAS-D

Anxiety symptoms were assessed using the German version of the Spence Children’s Anxiety Scale (SCAS-D; [25]. The 38 SCAS-D items refer to the major subtypes of DSM-IV anxiety disorders including separation anxiety (6 items), social phobia (6 items), obsessive-compulsive disorder (6 items), panic/agoraphobia (9 items), physical injury fears (5 items) and generalized anxiety disorder. Each item of the self-administered questionnaire is evaluated on a 4-point Likert scale, indicating the frequency of occurrence ranging from 0 (“never”) to 3 (“always”). Both the total score and subscale scores have demonstrated exceptional validity and reliability in previous research [26]. Internal consistency in our sample was excellent (*α* = 0.94) for the SCAS-D total score.

#### SCOFF

ED-symptoms were measured with the German version of the SCOFF questionnaire (Sick, Control, One stone, Fat, Food) [27]. The 5-item measure is considered a highly effective screening tool for eating disorders and is commonly used in institutions for general health [28]. The self-report items are rated on a nominal scale with responses categorized as either “yes” or “no”. Cronbach’s α in the current sample was 0.55.

### Procedure

Shortly after admission, patients of the LWL-University Hospital for Child and Adolescent Psychiatry Hamm took part in a routine diagnostic assessment including a wide variety of questionnaires, for example, the PHQ-9, SCAS-D and SCOFF questionnaires. Data of patients who were admitted and took part in routine diagnostics between 01/01/2019 and 23/02/2022 were used for the present study. The use of this data for study purposes was approved by the local medical-ethical committee of the Ruhr-University Bochum (No.: 4359-12).

### Data Preparation and analysis

Only participants who filled in all three questionnaires of interest were included in the study. After data preparation, descriptive statistics, t-tests and chi^2^-tests were calculated using IBM SPSS statistics (version 29.0.2.0 (20)). Network analyses were conducted using R (version 4.3.1) and RStudio (version 2023.06.1 + 524). There were no missing data in the study set.

### Network Estimation

Regularized partial-correlation networks were computed for both the pre pandemic (*n* = 596) and the peri pandemic (*n* = 765) sample including nodes of depression, anxiety, eating disorder and the covariates age and gender. Networks were estimated using the ‘graphical least absolute shrinkage and selection operator’ (*glasso*) function of the *qgraph* package in R to attenuate potential spurious links [29, 30]. The Extended Bayesian Information Criterion (EBIC) with a tuning parameter (γ) of 0.25 was chosen to identify the most accurate network [31, 32]. Spearman correlation was applied to prevent an overestimation of connections. The most common aspects of centrality (strength, closeness, betweenness and expected influence; [18]) were calculated using the *centralityPlot* and *centralityTable* functions in the *qgraph* package [29].

### Network stability

Network stability in both samples was tested using the *case-dropping* function of the *bootnet* package in R [33]. Stability of edge-weight accuracy, stability of centrality indices and of corresponding centrality stability coefficients (SCs) were estimated. SCs represent the percentage of cases that can be dropped from a sample while sustaining equal correlation values between the initial samples’ centrality indices and the bootstrapped samples’ centrality indices. To evaluate centrality accurately, a stability coefficient above 0.25, ideally above 0.50 is recommended [33].

### Network comparison

To identify differences in network density between the pre pandemic and the peri pandemic sample a Network Comparison Test (NCT) was conducted using the *NetworkComparisonTest* package in R [20]. The NCT is performed to detect whether the way the nodes within each network are connected differs across samples (‘Network Structure Invariance’) and whether the summed edge weights of the networks differ across samples (‘Global Strength Invariance’) [20]. Additionally, we examined centrality invariance of nodes between the two networks performing the Centrality Invariance Test within the NCT.

### Bridge symptoms

Bridge symptoms between anxiety, depression and ED within the two networks were identified using the *bridge* function of the *networktools* package in R [34]. Bridge centrality metrics include bridge strength and bridge expected influence (BEI). Bridge strength is determined by adding the absolute values of all edges connecting a certain node within a cluster with all other nodes of a different cluster. Calculating bridge strength reveals which symptom of one community of symptoms is most strongly connected to all symptoms of a different community. BEI represents the total of partial correlations between a certain node and all other nodes that are not in the same cluster, accounting for positive as well as negative correlations. Consequently, high BEI values indicate a mainly positive connection of a node to other nodes [35].

## Results

### Descriptive statistics

An overview of the sample’s characteristics and overall levels of depression, anxiety and ED are displayed in Table 1. Due to statistically significant differences in gender distribution between groups, we decided to control for age and gender in the network analysis, similarly to Armour et al. [36].

**Table 1:** Sample characteristics for the two groups

Variable	Pre COVID (*n* = 596)	Peri COVID (*n* = 765)	*t* statistic	Cohen’s *d*
	*M* (SD)	*M* (SD)		
Age	15.40 (1.44)	15.25 (1.49)	1.781	0.097
Treatment duration	54.72 (37.34)	54.86 (37.73)	− 0.065	− 0.004
No. of diagnoses	2.41 (1.34)	2.32 (1.43)	1.232	0.067
Depressive symptoms^*a*^	13.11 (6.44)	14.02 (6.84)	− 2.521*	− 0.137
Anxiety symptoms^*a*^	41.01 (19.36)	42.79 (20.80)	− 1.633	− 0.088
ED symptoms^*a*^	1.56 (1.32)	1.69 (1.41)	− 1.829	− 0.099

	*n* (%)	*n* (%)	Chi^2^	Cohen’s *d*
Female	352 (59.1)	501 (65.5)	*χ*^2^ = 5.920*	−0 0.151
Comorbidities	435 (73.0)	501 (65.5)	*χ*^2^ = 8.766**	0.195
Primary diagnosis^b^				
Depressive disorders	272 (45.6)	370 (48.4)	*χ*^2^ = 1.001	− 0.061
Conduct disorders	148 (24.8)	144 (18.8)	*χ*^2^ = 7.177**	0.195
Substance abuse disorders	79 (13.3)	110 (14.4)	*χ*^2^ = 0.354	− 0.052
Other	97 (16.3)	141 (18.4)	*χ*^2^ = 1.079	− 0.083
Depressive disorders^c^	410 (68.8)	492 (64.3)	*χ*^2^ = 3.006	0.111
Anxiety disorders^c^	76 (12.8)	107 (14.0)	*χ*^2^ = 0.439	− 0.059
Eating disorders^c^	19 (3.2)	52 (6.8)	*χ*^2^ = 8.827**	− 0.438

Group differences were tested with t-tests and Chi^2^-tests

^a^Total score of the respective questionnaire. Depression was measured with the PHQ-9, Anxiety with the SCAS-D and Eating Disorder symptoms with the SCOFF

^b^Only diagnoses with a frequency above 10% in the current sample are reported in the table

^c^Main and secondary diagnoses of the three relevant diagnostic categories

***p < 0.001; **p < 0.01; *p < 0.05.

### Stability analyses

For the pre pandemic sample, stability of edge weights, strength and expected influence was good (*SCs* = 0.67, see Figure S.1 for the edge weight stability graph and Figure S.2 for the centrality stability graph). Stability of closeness was good (*SC* = 0.59) and stability of betweenness was acceptable (*SC* = 0.28). Stability of bridge strength and bridge expected influence was good (*SCs* = 0.59). In the peri pandemic sample, stability of edge weights, strength and expected influence in the peri pandemic sample was excellent (*SCs* = 0.75, see Figure S.3 for the edge weight stability graph and Figure S.4 for the centrality stability graph). Stability of closeness was good (*SC* = 0.60) and stability of betweenness was poor (*SC* = 0.21). Stability of bridge strength and bridge expected influence in the peri pandemic sample was excellent (*SCs* = 0.75). Since the centrality estimates of strength, expected influence, closeness and betweenness were substantially interrelated in both the pre pandemic network (*r* ≥.66) and the peri pandemic network (*r* ≥.57), we focused our interpretation on expected influence to increase readability and interpretability. We chose to report expected influence centrality over strength centrality.

### Differences in network density

The global strength invariance test within the NCT revealed a statistically significant difference (*p* = 0.006) between the pre pandemic (global strength = 22.65) and the peri pandemic (global strength = 24.58) sample, indicating that the ED-Anxiety-Depression network had greater density in the peri pandemic sample.

### Central nodes within the networks—expected influence

**Fig. 1:**
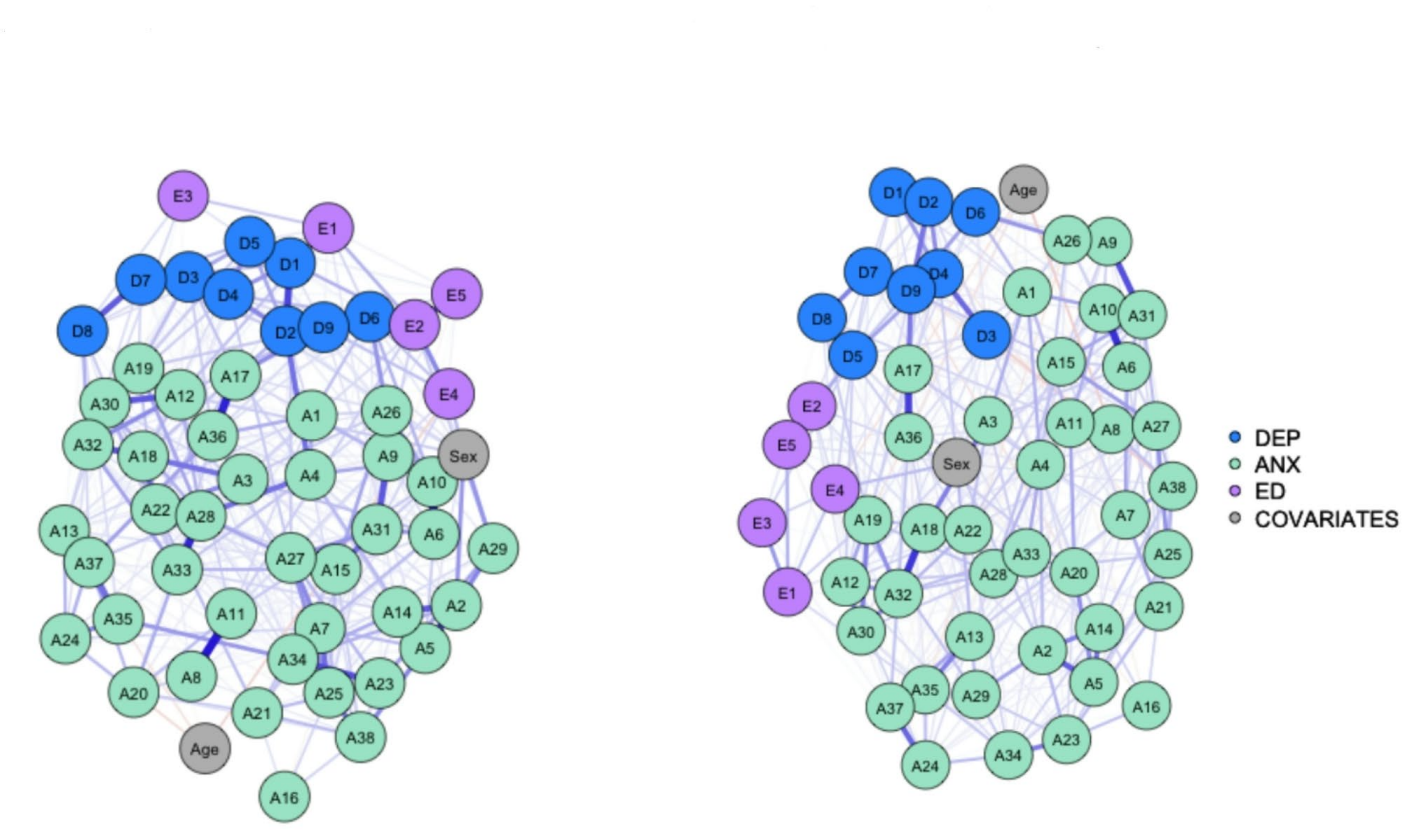
Networks of the pre pandemic and the peri pandemic sample. *Note.* Depressive symptoms are in blue, anxiety symptoms are in green, ED symptoms are in purple, and covariates are in gray. Thicker lines between nodes represent stronger relationships. Blue lines represent positive associations, red lines represent negative associations. Left panel: pre pandemic sample (*n* = 596), right panel: peri pandemic sample (*n* = 765). Network plots were created with the *plot* function of the *networktools* package [34] employing the *spring* layout which uses a Fruchterman–Reingold-force-directed algorithm

The networks (Fig. 1) consisted of 52 nodes (nine depression nodes [D1–D9], 36 anxiety nodes [A1–A36] and five ED nodes [E1–E5]), each representing a distinct item from the questionnaires employed in this study. Questionnaire nodes are displayed in Table S.1.

#### Pre pandemic network

**Fig. 2:**
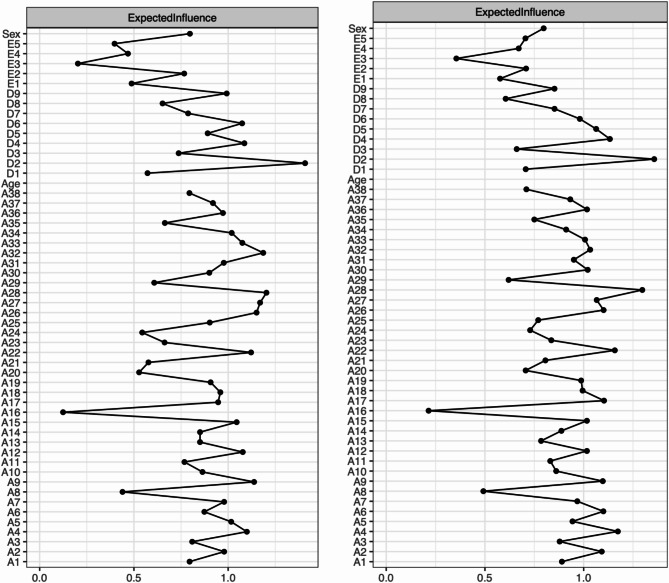
Expected influence centrality plots. Left panel: expected influence of the pre pandemic sample, right panel: expected influence of the peri pandemic sample. z-scores were used as the scale on the x-axis. Higher values indicate that a node is more central to the network

As can be seen in the left panel of Fig. 2, nodes with the highest expected influence within the pre pandemic network were feeling down, depressed, or hopeless [D2] (2.0), feeling scared for no reason [A28] (1.29), fast heartbeat for no reason [A32] (1.23), fear of crowded places/agoraphobia [A27] (1.18) and worry what other people think [A26] (1.11). Nodes with the lowest expected influence were fear of dogs [A16] (− 2.42) and recent weight loss [E3] (− 2.15).

#### Peri pandemic network

As can be seen in the right panel of Fig. 2, nodes with the highest expected influence within the peri pandemic network were feeling down, depressed, or hopeless [D2] (1.92), feeling scared for no reason [A28] (1.69), overall fear [A4] (1.21), feeling shaky when agonizing [A22] (1.15) and fatigue [D4] (1.05). Nodes with the lowest expected influence within the network were fear of dogs [A16] (− 2.54) and recent weight loss [E3] (− 1.99).

#### Differences in centrality between the networks

**Fig. 3:**
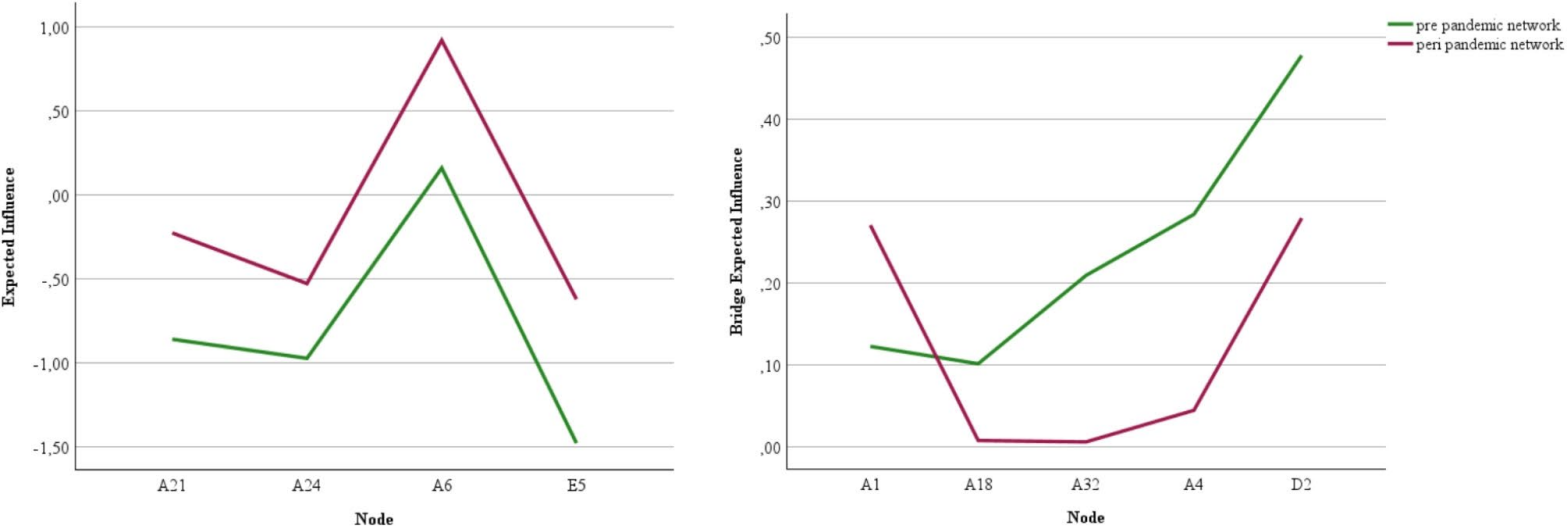
Significantly different expected influence and bridge expected influence values

The centrality invariance test revealed a significant difference (*p* < 0.05) of expected influence centrality in 4 of the 52 nodes (see Fig. 3, left panel). None of these nodes were among the most influential nodes mentioned above. All of the 4 nodes had a greater expected influence in the peri pandemic sample. Whereas the centrality values of fear of doctors [A21] (− 0.23), obsessive-compulsive thoughts [A24] (− 0.53) and food dominates life [E5] (− 0.62) were still relatively low in the peri pandemic sample, fear of tests at school [A6] had a relatively low expected influence in the pre pandemic sample (0.16) but reached a relevant centrality value in the peri pandemic sample (0.92).

### Central pathways within the networks—bridge symptoms

#### Pre pandemic network

BEI is displayed in the right panel of Fig. 2. In the pre pandemic network, feeling bad about yourself/ feeling of failure [D6] (BEI = 0.59), trouble getting rid of bad/ silly thoughts [A17] (BEI = 0.31) and self-induced vomiting [E1] (BEI = 0.31) were identified as the most influential bridge symptoms. Feeling bad about yourself/ feeling of failure from the depression community was most strongly connected to worry what other people think [A26] (part *r* = 0.13;) and body image disturbance [E4] (part *r* = 0.07) in the other communities. Trouble getting rid of bad/ silly thoughts was most strongly connected to suicidal thoughts [D9] (part *r* = 0.12) and did not correlate with any of the ED items. Self-induced vomiting was most strongly connected to feeling bad about yourself/feeling of failure [D6] (part *r* = 0.05) and sudden trouble breathing (part *r* = 0.07).

#### Peri pandemic network

**Fig. 4:**
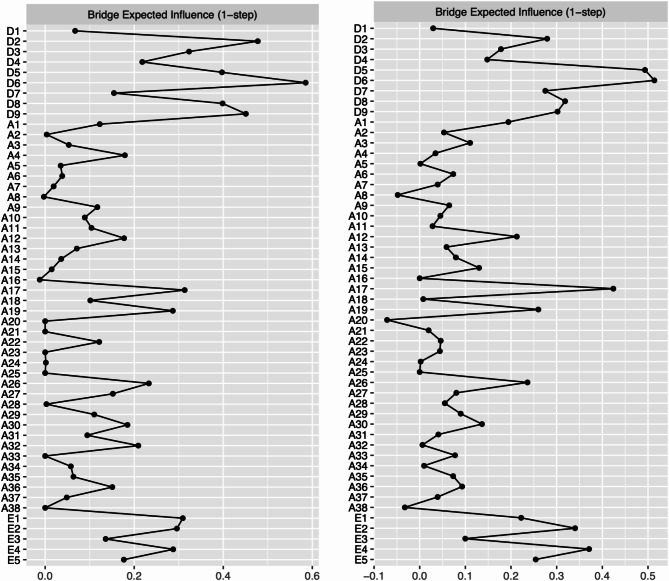
Bridge expected influence centrality plot. Left panel: Bridge Expected Influence of the pre pandemic sample, right panel: Bridge Expected Influence of the peri pandemic sample. z-scores were used as the scale on the x-axis. Higher values indicate that a node is more central to the network

Nodes with the highest BEI in the peri pandemic network are displayed in the right panel of Fig. 4. Feeling bad about yourself/ feeling of failure [D6] (BEI = 0.51), difficulties getting rid of bad/silly thoughts [A17] (BEI = 0.42) and body image disturbance [E4] (BEI = 0.39) were identified as bridge symptoms. Feeling bad about yourself/ feeling of failure from the depression community was most strongly connected to worry what other people think [A26] (part *r* = 0.16) and loss of control over food intake [E2] (part *r* = 0.04). Difficulties getting rid of bad/ silly thoughts was most strongly connected to suicidal thoughts [D9] (part *r* = 0.20) and no correlation was found with any of the ED items. Body image disturbance was most strongly connected to abnormal appetite [D5] (part *r* = 0.07) and fear of insects (part *r* = 0.09).

#### Differences in bridge symptoms between the networks

For BEI, the centrality invariance test revealed a significant difference between 5 nodes (see Fig. 3, right panel). However, none of these nodes were among the most influential nodes regarding BEI. Whereas heartbeat when agonizing [A18] (0.01), fast heartbeat for no reason [A32] (0.01), overall fear [A4] (0.04), and feeling depressed [D2] (0.28) had significant lower BEI during the pandemic as compared to before the pandemic, overall worry [A1] was observed to be higher in the peri pandemic sample (0.27 vs. 0.12).

## Discussion

The present study aimed to understand how the COVID-19 pandemic impacted networks of depression, anxiety, and ED symptoms. First, a Network Comparison Test was performed to examine both networks for differences in their density and overall connectivity (global strength). Subsequently, our focus was directed towards identifying the most central symptoms as well as symptoms that contributed to the occurrence of comorbidity. Significant changes in centrality and comorbidity were assessed between the networks.

### Network comparison

As hypothesized, the Network Comparison Test revealed that the networks differed in network density (global strength). Greater network density suggests an overall stronger connection between symptoms, indicating that symptoms in a denser network are more likely to interact with each other compared to those in a network with lower global strength [37]. For example, difficulties getting rid of bad/ silly thought or feelings of failure would be more prone to cause other ED, anxiety, and depression symptoms (e.g., suicidal thoughts) under the impact of the emotional burden caused by the pandemic due to stronger interconnectedness of symptoms. Our findings suggest that the ongoing of an impactful event, such as a global pandemic, is associated with an overall stronger network connectivity. These results support previous literature reporting about an increase in symptom severity among patients with pre-existing mental health conditions under the impact of the pandemic [13, 14].

### Core symptoms

In the pre pandemic sample, feeling down, depressed or hopeless, feeling scared for no reason, fast heartbeat, fear of crowded places and worry what other people think emerged as core symptoms with the highest expected influence within the network. Thus, depression and anxiety seemed to be most central, while ED-symptoms were more peripheral.

In the peri pandemic sample, feeling down, depressed or hopeless and feeling scared for no reason persisted as symptoms with the highest expected influence. In addition, overall fear, fatigue and feeling shaky when agonizing emerged as core symptoms. Thus, depression and anxiety symptoms seemed to be most central, ED-symptoms had less importance for the network. The imbalance of diagnoses in the sample (> 50% diagnosed with depression, < 10% diagnosed with anxiety or ED) may have contributed to ED-symptoms being more peripheral in the network. It should be noted that anxiety symptoms were central to the network despite the lower number of individuals diagnosed with anxiety. This is likely due to a stronger association between anxiety symptoms and depression symptoms. Our findings are in line with other network analyses describing sad mood, low energy and inability to control worry as symptoms most central to depression and anxiety networks [38, 39]. Our results show that during the COVID-19 pandemic, there were no significant differences between samples regarding the expected influence values of the 5 most important core symptoms. However, we observed a shift in the relative ranking of the nodes. Fear of crowded places and worry what other people think did not change significantly between samples, highlighting their consistency as core symptoms. Yet, their position in the overall ranking of most influential symptoms decreased. Despite the evidence of increased social anxiety and agoraphobia levels among adolescents during the pandemic [40, 41], these symptoms appear to have been outranked by other core symptoms. Our findings indicate that the relative importance of various mental health concerns shifted during the pandemic. In the peri pandemic sample overall fear, fatigue and feeling shaky when agonizing emerged as additional core symptoms. These results reflect study results indicating that during the pandemic adolescents were particularly affected by sleep disturbances caused by later bedtimes, fear of infection, loss of family members or friends, concerns over consequences of social restriction and uncertainty about the future [42, 43]. Yet, it should be noted, that the changes between the networks regarding the most influential nodes were non-significant and thus rather descriptive in nature. A significantly higher expected influence was observed for four nodes, all of which were not among the most influential nodes. While three out of four nodes were still irrelevant in the peri pandemic network, fear of tests at school gained a relatively strong expected influence within the peri pandemic sample. This aligns with recent findings detecting high levels of online exam anxiety and fear of educational failure among students during the pandemic [44, 45].

### Bridge symptoms

Bridge centrality statistics measure the connectivity among various communities of nodes and shed light on relationship patterns between symptoms of comorbid disorders. Difficulties getting rid of bad/ silly thoughts (ANX) and suicidal thoughts (DEP) emerged as the strongest pathway between depression and anxiety symptoms in the pre as well as the peri pandemic sample. This suggests that the bridge between experiencing difficulties getting rid of bad/silly thoughts and suicidal thoughts represents a stable pathway between depression and anxiety disorder, potentially driving comorbidity and highlighting its relevance for clinical interventions. Another pathway that persisted across samples was the bridge that evolved between feeling bad about yourself/ feelings of failure (DEP) and worry what other people think (ANX). Additionally, feeling bad about yourself/feeling of failure was the symptom with the highest BEI in both samples. The persistence of the mentioned connections between symptom clusters of depression and anxiety after the onset of the pandemic suggests that those pathways are of high clinical relevance between the disorders, independent of the event, and should be considered in treatment. These findings are backed by the centrality invariance test that revealed no significant differences in bridge expected influence centralities of these symptoms between the samples. On a different note, the challenges and disruptions brought about by the COVID-19 pandemic were associated with changes of certain pathways. For example, the link between feeling bad about yourself/ feeling of failure (DEP) and loss of control over food intake (ED) was among the most prominent pathways in the peri pandemic sample. This is in line with existing literature on the pandemic’s impact on eating disorder patients, who experienced a loss of control over eating habits due to various factors such as limited access to certain foods, disruptions in treatment and an overall lack of routine and structure [46–48]. However, for some patients, control over their eating habits increased during lockdown [49]. It is important to note that body image disturbance, which was linked to feeling bad about yourself/ feelings of failure in the pre pandemic sample, did not disappear as a bridge component in the peri pandemic sample. In fact, body image disturbance replaced self-induced vomiting as the most influential bridge symptom among the ED symptoms. Body image disturbance was most strongly linked to abnormal appetite (DEP) which is consistent with the literature highlighting increased or decreased appetite and overall worsened ED symptoms among patients with ED during lockdown [13, 50]. Body image disturbance also formed a bridge with fear of insects (ANX) which may be understood in the light of increased health-related worries under the influence of the pandemic [51]. A higher focus on health and hygiene may have intensified concerns about potential health threats, including insects. Further, studies found one’s perceived health status to be associated with avoidance behavior towards insects [52]. Along with increased health-related worries, this may have contributed to the connection between body image issues and fear of insects. It should be noted that although there were links between ED symptoms and other communities, these connections were relatively weak. This is likely since ED symptoms were generally more peripheral in the network. It is worth noting, however, that the centrality invariance test showed no significant difference between the samples for the most influential bridge symptoms. This underlines our finding that comorbidity pathways seem to remain stable even under specific circumstances such as a global pandemic. A further result of the centrality invariance test is a significant change in the importance of five symptoms between networks. Fast heartbeat for no reason [A32], heartbeat when agonizing [A18], overall fear [A4] and feeling down, depressed or hopeless [D2] had lower bridge values within the peri pandemic. This indicates that these symptoms were less important for driving comorbidity during the pandemic as compared to before the pandemic. In contrast, overall worry [A1] showed a significantly higher bridge expected influence during the pandemic, suggesting that, rather than serving as a bridge symptom specific to anxiety disorder, it may represent a key node promoting comorbidity under the ongoing of a major disruptive event. These findings emphasize the importance of identify bridge symptoms among comorbid disorders to be able to optimize therapeutic interventions and target symptom connections that may drive comorbidity.

### Translation to psychotherapy

Understanding the concept of comorbidity has always been a challenge in psychopathology. Identifying bridge symptoms through network analysis is important because our results show that most bridge symptoms were relatively stable between networks. Bridge symptoms tend to activate a comorbid networks, even if they appear less prominent in the network at first glance. These findings are coherent with the suggestion that symptoms associated with comorbidity are not necessarily the symptoms considered most specific for a disorder [18]. With regard to psychotherapy, core symptoms of a disorder should be targeted to achieve an overall disruption of a symptom network. However, targeting bridge symptoms may effectively influence underlying mechanisms that promote comorbidity between disorders. The current data then point to a high relevance of feeling down, depressed or hopeless, feeling scared for no reason overall fear, feeling shaky when agonizing and fatigue as core symptoms during the pandemic. The current data also highlight the pathways between feeling bad about yourself/ feelings of failure (DEP) and worry what other people think (ANX) as well as between difficulties getting rid of bad/ silly thoughts (ANX) and suicidal thoughts (DEP). These connections may play a key role in maintaining comorbidity, thus serving as primary targets for intervention in patients with comorbidities, regardless of any ongoing impactful event.

### Limitations

There are several limitations to acknowledge in this study. Of note, the sample did not exhibit an equal balance across the three categories of depression, anxiety and ED. While 53.1% of the sample had a diagnosis of depression, only a small group of patients (< 10%) were diagnosed with anxiety or ED. This might lead to an underrepresentation of anxiety and ED symptoms. In addition, the sample was highly comorbid with a wide range of psychopathologies. Symptoms were assessed through self-report and might be biased by the emotional state. Cronbach’s α for the SCOFF questionnaire total score was low in the current study. However, in the original validation study by Morgan et al. [27], the author concluded that the SCOFF appears to be a highly effective screening tool for detecting EDs. The low internal consistency of the questionnaire can be explained by EDs being a rather multidimensional concept. Additionally, while previous research highlights the association of family conflicts and impulsivity traits with eating disorder psychopathology in the context of COVID-19 [53], our study design did not account for pandemic-specific shifts (e.g. family conflicts, disruptions to daily routines, loss of independence and diminished social connections). We followed standard practice in network analysis research by controlling for age and gender but acknowledge that other potential covariates were not available in our dataset. Future studies conducting network analyses should incorporate such variables to provide further insight into their role in shaping symptom networks and gain a clearer understanding of the drivers behind symptom changes during the pandemic. A more in-depth exploration of these psychosocial aspects could offer valuable insight into their role in the occurrence of comorbidities. A promising direction for future research could involve combining the design of the study by Ioannidis et al. (2022) [53] and the current study, investigating pre- and peri pandemic networks within the same sample, including not only symptom nodes but also sociodemographic variables, personality traits, and family interaction patterns. Furthermore, we did not account for medication status which may have had a profound impact on symptom networks. Bos et al. [54] investigated the influence of antidepressant medication on patients with major depressive disorder by performing network analyses on cross-sectional depression networks before and after treatment. A significant reduction in depressive symptom severity was observed between samples. Yet, they suggest that this observation is more likely related to increased symptom interconnectivity (network density) rather than by medication status itself. However, future studies would benefit from accounting for factors such as medication status as well as treatment history and hospitalization criteria to explore their potential influence on symptom networks. As we used cross-sectional data for this study, we cannot make any statements regarding causality and predictive values of our findings. Observed changes in symptom networks should not be interpreted as direct effects of the pandemic but rather as associations that warrant further investigation using longitudinal designs. Additionally, we cannot determine whether the observed changes reflect natural fluctuations or were directly caused by the COVID-19 pandemic. Prior research suggests that symptoms tend to vary over time and do not always remain consistent [55]. Finally, this study utilized a developmental sample limited to adolescents ages 13 to 21. Therefore, findings may not be directly applicable to other demographic groups. Future studies should conduct network analyses across a broader spectrum of age groups to enhance the generalizability of the results to a wider range of patients with different demographic characteristics. Analyzing and comparing two groups that are more consistent in sample characteristics, for example in a unimorbid depressive sample, would also help to validate the findings of our study, ensuring that our results are not driven by specific subgroups.

## Conclusion and future directions

This was the first study to compare comorbid depression-anxiety-ED-networks before and during the onset of the COVID-19 pandemic. We aimed to investigate changes in the relationship between depression, anxiety and ED highlighting central as well as bridge symptoms and comparing the networks regarding the symptoms’ interconnectedness. Our findings suggest a multidimensional interrelation between various aspects of depression, anxiety, and EDs. The persistence of bridges between the networks throughout the pandemic affirms their validity and implicates that patients would benefit from treatment that focuses on weakening those specific connections. The statistically significant difference in network density between networks highlights the importance of investigating changes in symptom networks in response to impactful events such as a global pandemic. Intervention strategies might require adjustment to address the evolving needs of adolescent inpatients. Future studies should investigate the applicability of our findings to psychiatric and psychotherapeutic interventions. Future studies should also conduct network analysis on longitudinal data (e.g., network density of a post COVID network) to investigate changes in symptom networks over time. This would provide insight into potential causal pathways and enhance the predictive power of the analysis.

## Electronic supplementary material

Below is the link to the electronic supplementary material.

Supplementary Material 1

## Data Availability

No datasets were generated or analysed during the current study.

## Abbreviations

COVID-19: Corona Virus Disease 2019
DEP: Depression
ANX: Anxiety
ED: Eating disorder
PHQ-9: Patient Health Questionnaire
SCAS-D: Spence Children’s Anxiety Scale
SCOFF: Sick, control, one stone, fat, food
GLASSO: Graphical Least Absolute Shrinkage and Selection Operator
EBIC: Extended Bayesian Information Criterion
SC: Stability coefficient
NCT: Network comparison test
BEI: Bridge expected influence
