# Supplementary material for: Understanding the impact of COVID-19 on comorbid depression, anxiety and eating disorders in adolescent psychiatric inpatients: a network analysis
Source: Child Adolesc Psychiatry Ment Health. 2025 Apr 23;19:44. doi: 10.1186/s13034-025-00899-0 (PMC12020227; doi:10.1186/s13034-025-00899-0)
Supplement: Supplementary file 1 — Supplementary Material 1 [file 13034_2025_899_MOESM1_ESM.docx]

**Supplementary Material**

**Figure S.1**

*Edge Weights Stability in the Pre Pandemic Sample*

*
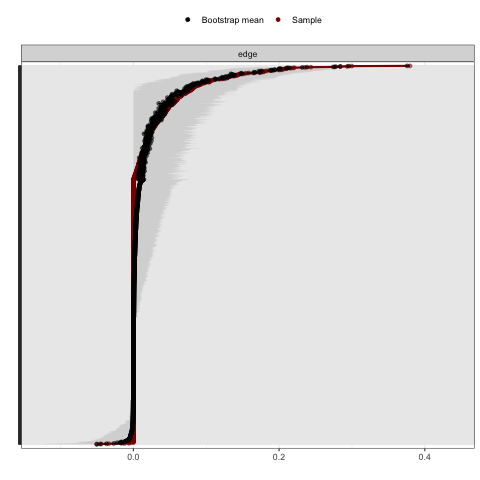
*

*Note.* Bootstrapped confidence intervals of estimated edge-weights for the pre pandemic network. The red line indicates the sample values, and the gray area indicates the bootstrapped confidence intervals. Each horizontal line represents one edge of the network, ordered from highest to lowest edge-weight.

**Figure S.2**

*Centrality Stability in the Pre Pandemic Sample*

***
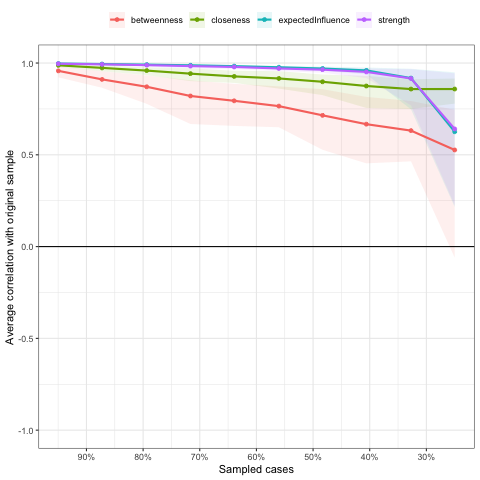
***

*Note.* The x-axis reports the average correlations between centrality indices of the original sample and the centrality indices from re-estimated networks after dropping cases. Lines indicate the correlations of centrality indices and *areas* indicate the range from the 2.5th quantile to the 97.5th quantile.

**Figure S.3**

*Edge Weights Stability in the Peri Pandemic Sample*

*
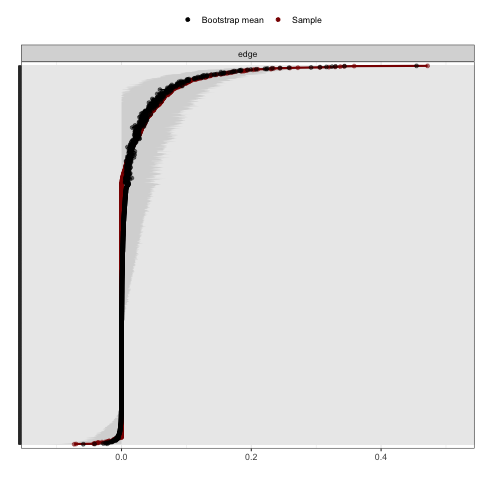
*

*Note.* Bootstrapped confidence intervals of estimated edge-weights for the peri pandemic network. The red line indicates the sample values, and the gray area indicates the bootstrapped confidence intervals. Each horizontal line represents one edge of the network, ordered from highest to lowest edge-weight.

**Figure S.4**

*Centrality Stability in the Peri Pandemic Sample*

**
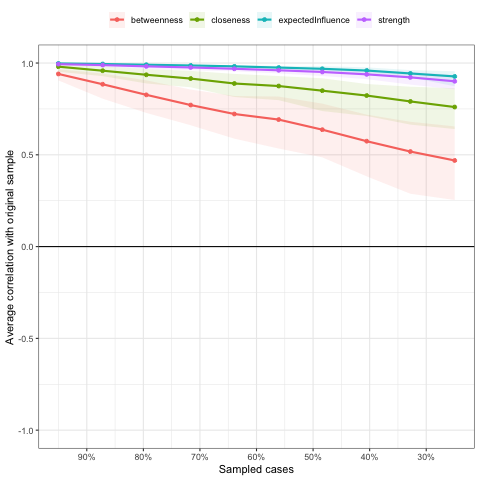
**

*Note.* The x-axis reports the average correlations between centrality indices of the original sample and the centrality indices from re-estimated networks after dropping cases. Lines indicate the correlations of centrality indices and *areas* indicate the range from the 2.5th quantile to the 97.5th quantile.

**Table S.1**

*Nodes in the Networks*

| Abbreviation | Item | Node |
| --- | --- | --- |
| Loss of interest | Little interest or pleasure in doing things | D1 |
| Feeling depressed | Feeling down, depressed, or hopeless | D2 |
| Sleeping problems | Trouble falling or staying asleep or sleeping too much | D3 |
| Fatigue | Feeling tired or having little energy | D4 |
| Abnormal appetite | Poor appetite or overeating | D5 |
| Feeling bad about yourself/ feeling of failure | Feeling bad about yourself – or that you are a failure or have let yourself or your family down | D6 |
| Difficulties concentrating | Trouble concentrating on things, such as reading the newspaper or watching television | D7 |
| Psychomotoric retardation/ agitation | Moving or speaking so slowly that other people could have noticed or the opposite - being so fidgety or restless that you have been moving around a lot more than usual | D8 |
| Suicidal thoughts | Thoughts that you would be better off dead or of hurting yourself in some way | D9 |
| Overall worry | I worry about things | A1 |
| Fear of darkness | I am scared of the dark | A2 |
| Funny feeling in stomach | When I have a problem, I get a funny feeling in my stomach | A3 |
| Overall fear | I feel afraid | A4 |
| Fear of being alone | I would feel afraid of being on my own at home | A5 |
| Fear of tests at school | I feel scared when I have to take a test | A6 |
| Fear of public facilities | I feel afraid if I have to use public toilets or bathrooms | A7 |
| Fear of being away from parents | I worry about being away from my parents | A8 |
| Fear of embarassment | I feel afraid that I will make a fool of myself in front of people | A9 |
| Worry over school performance | I worry that I will do badly at my school work | A10 |
| Worry over family members | I worry that something awful will happen to someone in my family | A11 |
| Sudden trouble breathing | I suddenly feel as if I can’t breathe when there is no reason for this | A12 |
| Repeated checking behavior | I have to keep checking that I have done things right | A13 |
| Fear of sleeping alone | I feel scared if I have to sleep on my own | A14 |
| Trouble going to school | I have trouble going to school in the mornings because I feel nervous or afraid | A15 |
| Fear of dogs | I am scared of dogs | A16 |
| Trouble getting rid of bad/ silly thoughts | I can’t seem to get bad or silly thoughts out of my head | A17 |
| Heartbeats when agonizing | When I have a problem my heart beats really fast | A18 |
| Shaking for no reason | I suddenly start to tremble or shake when there is no reason for this | A19 |
| Worry over myself | I worry that something bad will happen to me | A20 |
| Fear of doctors | I am scared of going to the doctors or dentists | A21 |
| Shaky when agonizing | When I have a problem, I feel shaky | A22 |
| Fear of heights | I am scared of being in high places or lifts (elevators) | A23 |
| Obsessive-compulsive thoughts | I have to think of special thoughts to stop bad things from happening | A24 |
| Fear of public transport | I feel scared if I have to travel in the car, or on a bus or a train | A25 |
| Worry what other people think | I worry what other people think of me | A26 |
| Fear of crowding/ agoraphobia | I am afraid of being in crowded places | A27 |
| Scared for no reason | All of a sudden, I feel really scared for no reason at all | A28 |
| Fear of insects | I am scared of insects or spiders | A29 |
| Dizziness for no reason | I suddenly become dizzy or faint when there is no reason for this | A30 |
| Fear of speaking | I feel afraid if I have to talk in front of my class | A31 |
| Fast heartbeats for no reason | My heart suddenly starts to beat too quickly for no reason | A32 |
| Worry over becoming scared | I worry that I will suddenly get a scared feeling when there is nothing to be afraid of | A33 |
| Fear of small places | I am afraid of being in small, closed places, like tunnels or small rooms | A34 |
| Compulsive behavior | I have to do some things over and over again | A35 |
| Disturbed by bad thoughts | I get bothered by bad or silly thoughts or pictures in my mind | A36 |
| Obsessive-compulsive actions | I have to do some things in just the right way to stop bad things happening | A37 |
| Fear of staying away over night | I would feel scared if I had to stay away from home over night | A38 |
| Self-induced vomiting | Do you make yourself sick because you feel uncomfortably full? | E1 |
| Loss of control over food intake | Do you worry you have lost control over how much you eat? | E2 |
| Recent weight loss | Have you recently lost one stone in a 3-month period? | E3 |
| Body image disturbance | Do you believe yourself to be fat when others say you are too thin? | E4 |
| Food dominates life | Would you say that food dominates your life? | E5 |

*Note.* Nodes D1-D9 represent items of the PHQ-9 questionnaire, nodes A1-A38 represent items of the SCAS-D questionnaire and nodes E1-E5 represent items of the SCOFF questionnaire.
